# Supplementary material for: Orbital and millennial-scale forcing of the Patagonian Ice Sheet throughout the Last Glacial Cycle
Source: Nat Commun. 2025 Oct 2;16:8776. doi: 10.1038/s41467-025-64614-5 (PMC12491557; doi:10.1038/s41467-025-64614-5)
Supplement: Supplementary file 1 — Supplementary Information [file 41467_2025_64614_MOESM1_ESM.pdf]

Supplementary Information

Orbital and Millennial-scale forcing of the Patagonian ice sheet throughout the Last Glacial Cycle.

Castillo-Llarena, Andrés [1*]; Prange, Matthias [1]; Rogozhina, Irina [2,3,4]

[1] MARUM- Center for Marine Environmental Sciences and Faculty of Geosciences, University of Bremen, Bremen, Germany

[2]Centro de Estudios Avanzados en Zonas Áridas (CEAZA), La Serena, Chile

[3]Department of Geography, Norwegian University of Science and Technology, Trondheim, Norway

[4]University of La Serena, Faculty of Enginieering, La Serena, Chile

[*] acastillollarena@marum.de

This PDF file includes:

Supplementary Table 1
Supplementary Figures 1-6
Supplementary References

Supplementary Table 1**. Key parameters of the model setup.**

Description	Values	Units
Ice density	910.0	$kg\ m^{-3}$
Gravity acceleration	9.81	$m\ s^{-1}$
Glen's flow law exponent	3.0	-
Scaling reference speed for hybrid SIA and SSA dynamics	10.0	$m\ a^{-1}$
Sliding coefficient	10.0	$m\ a^{-1} Pa^{-1}$
Sub-melt sliding coefficient	3.0	K
Sliding exponents (p,q)	3, 2	-
Time lag of the relaxing asthenosphere	3000	a
Flexural rigidity of the lithosphere	10^{25}	Nm
Geothermal heat flux	100	$mW\ m^{-2}$
Lithosphere density	3300	$kg\ m^{-3}$
PDD standard deviation	3	$ºC$
Temperature of snow precipitation	0	$ºC$
Temperature of rain precipitation	2	$ºC$
Degree day factor for snow	6	$mm\ d^{-1} ºC^{-1}$
Degree day factor for ice	3	$mm\ d^{-1} ºC^{-1}$

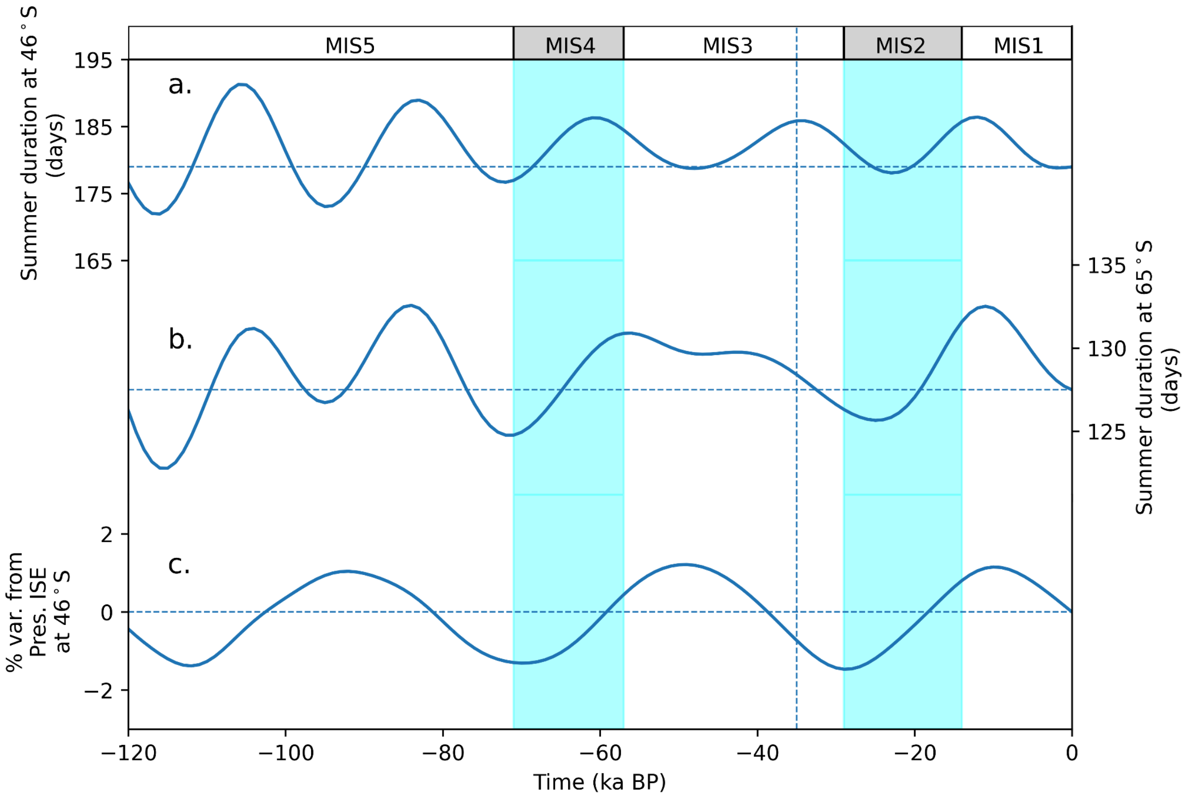

Supplementary Figure 1**. Comparison of summer duration and integrated summer energy**. a. summer duration in days at 46ºS and b. summer duration in days at 65ºS, filtered [1]. c. Percentage of variation of the integrated summer energy at 46ºS compared to present day. Threshold for both summer duration and integrated summer energy is set to 300 Wm^{-2}. Horizontal dashed lines indicate the present day values for each subplot as reference. Vertical line indicates the geologically constrained maximum extension in Patagonia at 35 ka [2].

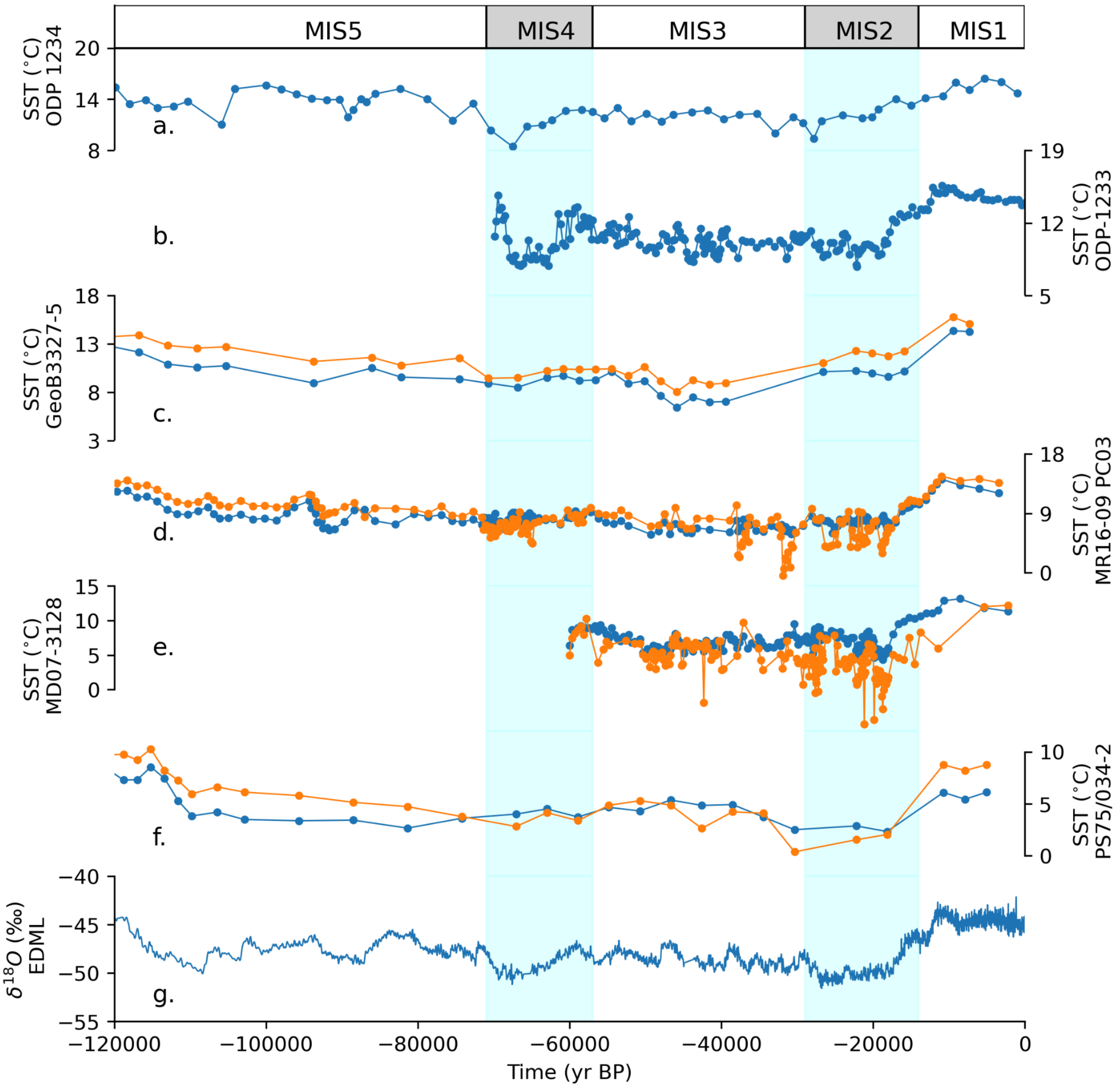

Supplementary Figure 2. **Sea surface temperature reconstructions. a-f** Sea surface temperatures of additional cores along the Chilean margin [3–9]. Orange lines show Uk37 and blue lines indicate Uk'37. **g** $\delta^{18}O$ record from the EDML ice core [10].

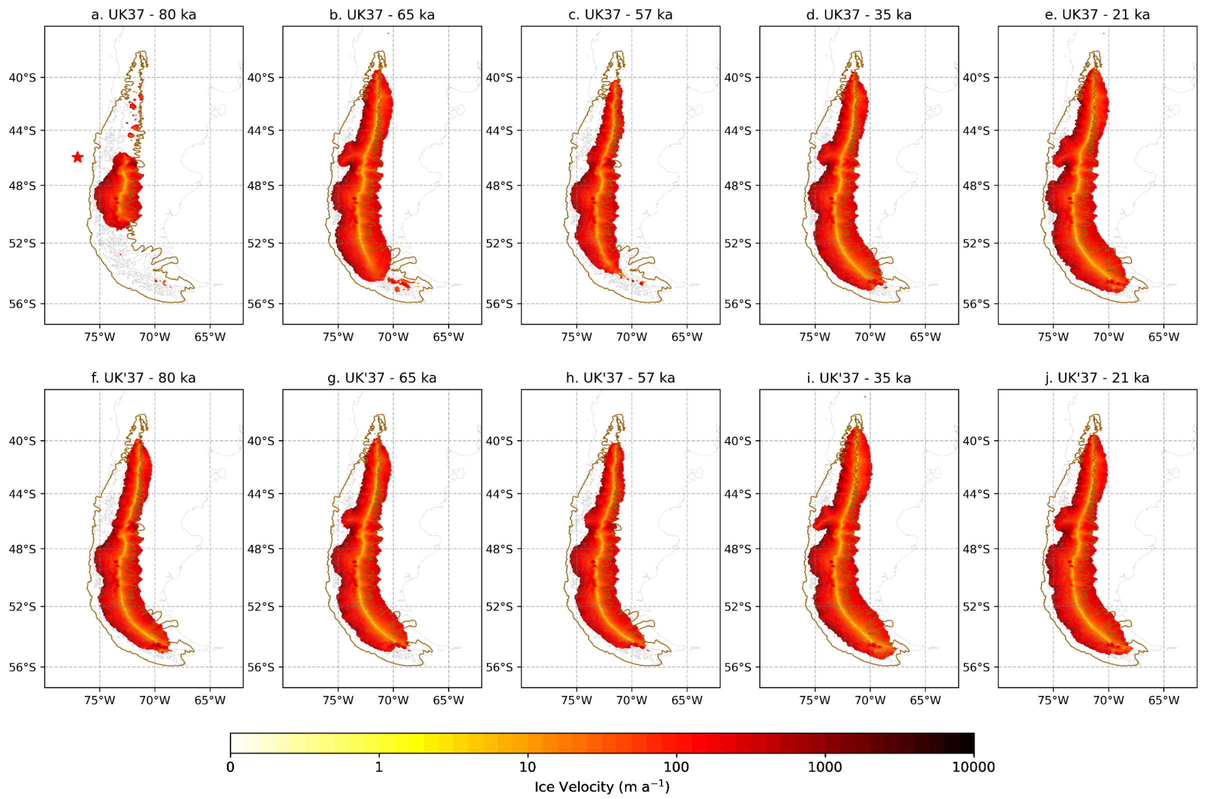

Supplementary Figure 3**. Ice sheet velocity**. Ice velocities modelled at 80, 65, 57, 35 and 21 ka by using the glacial index method using the calibration Uk37 (upper panels) and Uk'37 (bottom panels) as representatives of the MIS5, MIS4, early MIS3, MIS3-MIS2 transitions and LGM, respectively. The geochronological reconstruction of the PIS at 35 ka is shown in brown [2]. Red star indicates the position of the marine sediment core MR16-09 PC03 [11].

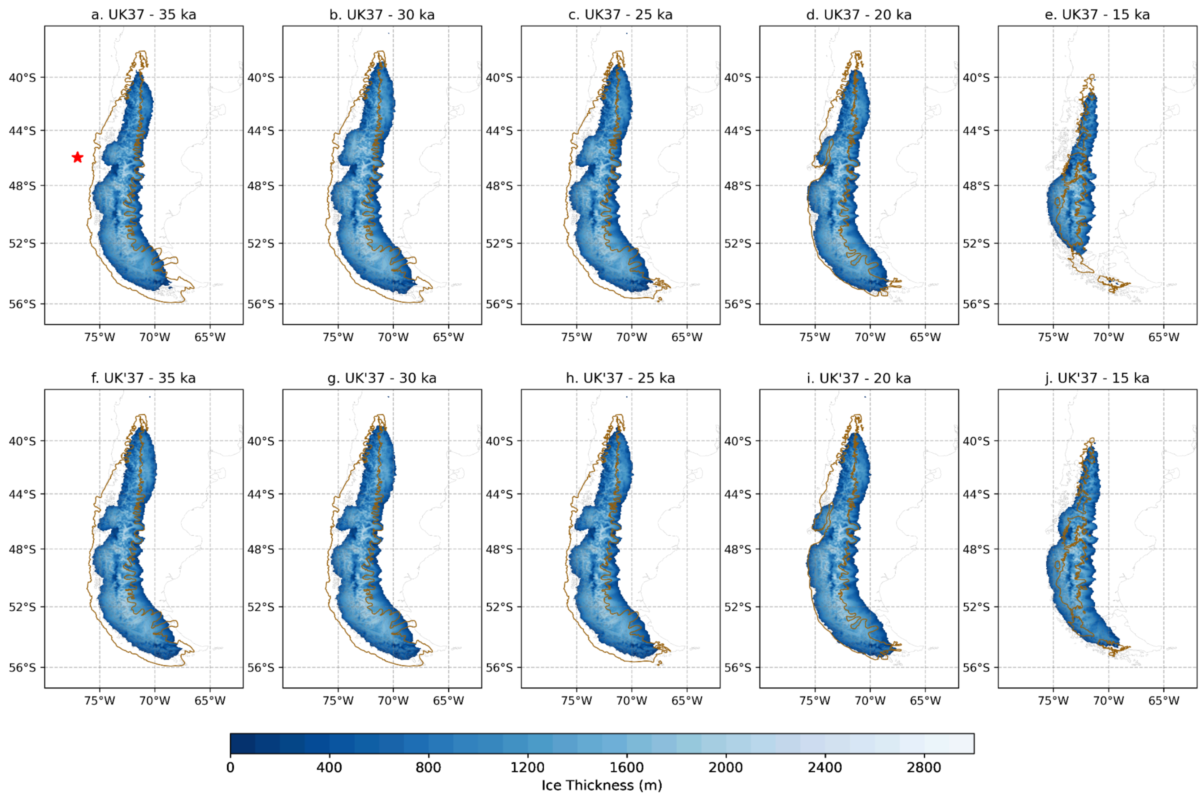

Supplementary Figure 4. **Ice sheet geometry during the deglaciation.** Ice thickness modelled at 35, 30, 25, 20 and 15 ka by using the glacial index method using the calibration Uk37 (upper panels) and Uk'37 (bottom panels) as representatives of the MIS5, MIS4, early MIS3, MIS3-MIS2 transitions and LGM, respectively. The geochronological reconstruction of the PIS at each time slide is shown in brown [2]. Red star indicates the position of the marine sediment core MR16-09 PC03 [11].

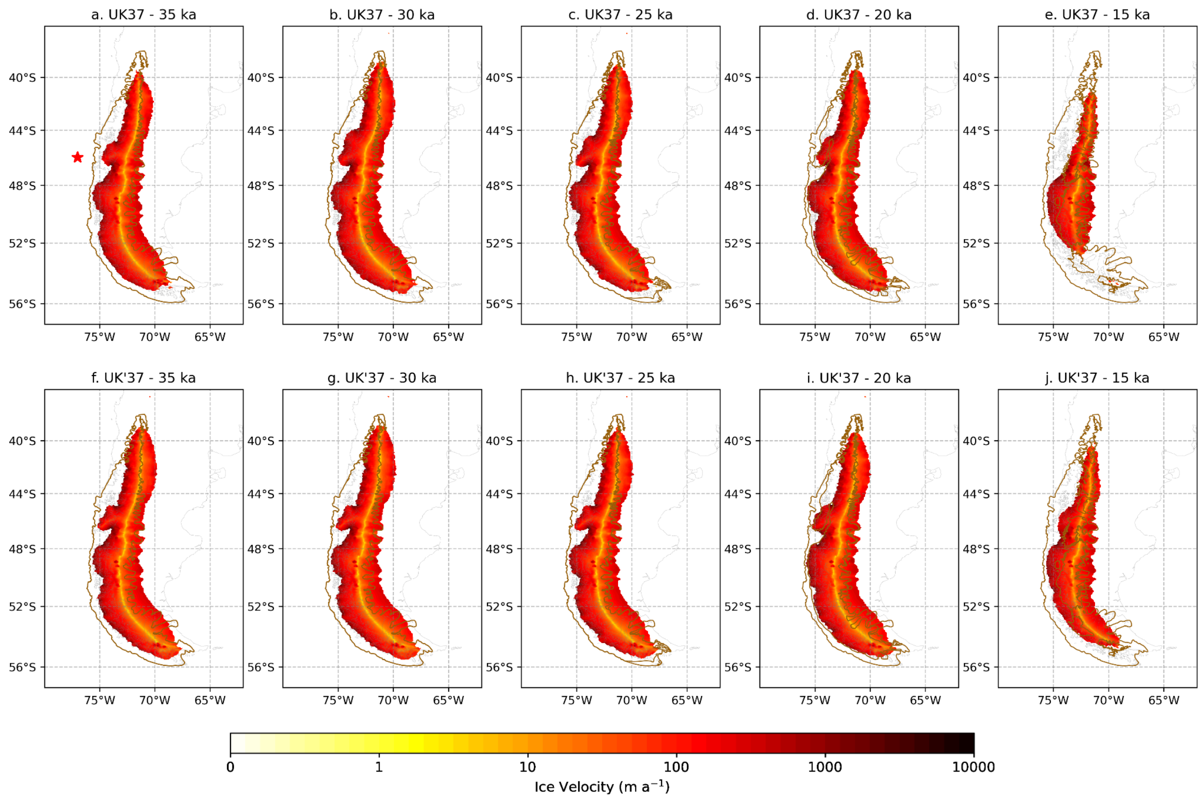

Supplementary Figure 5. **Ice sheet Velocity during the deglaciation. a-e** Ice velocities modelled at 35, 30, 25, 20 and 15 ka by using the glacial index method using the calibration Uk37. **f-j** Ice velocities modelled at 35, 30, 25, 20 and 15 ka by using the glacial index method using the calibration Uk'37. Time slices are chosen as representatives of the MIS5, MIS4, early MIS3, MIS3-MIS2 transitions and LGM, respectively. The geochronological reconstruction of the PIS at each time slide is shown in brown [2]. Red star indicates the position of the marine sediment core MR16-09 PC03 [11].

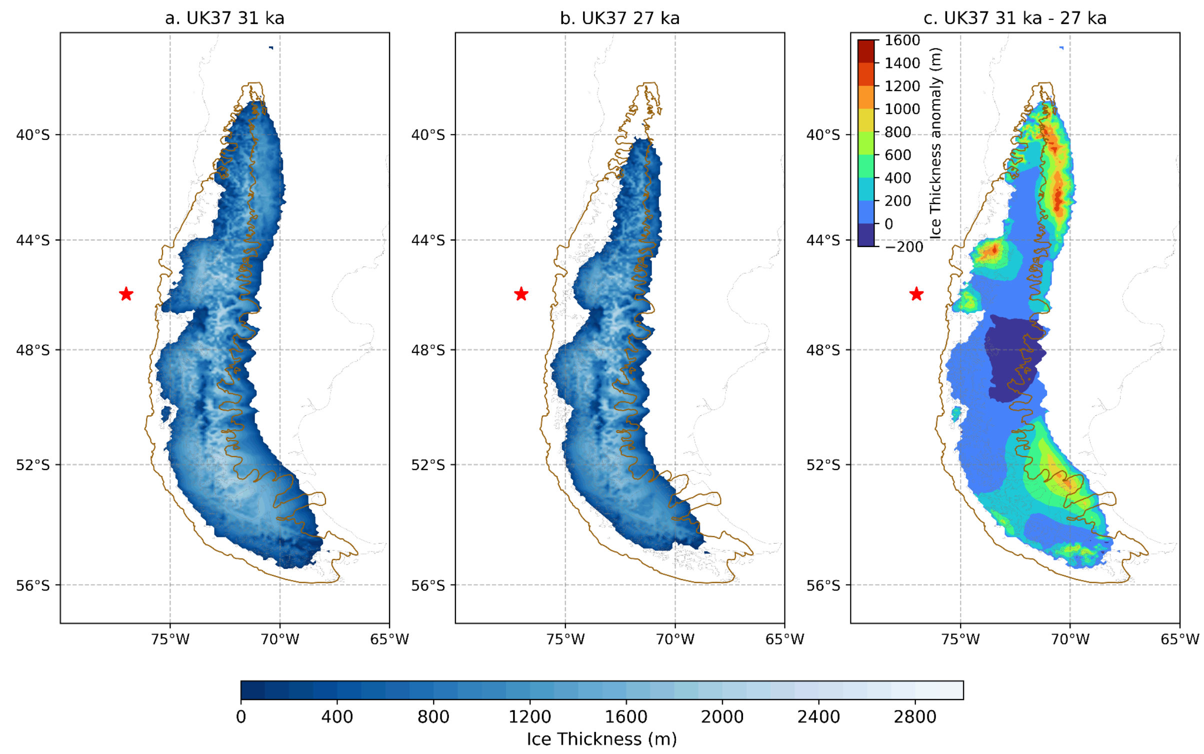

Supplementary Figure 6. **Ice sheet thickness anomaly under millennial-scale variability.** Ice shee thickness modelled at 31 and 27 ka by using the glacial index method using the calibration Uk37 as representatives of relative maximum and minimum of the millennial-scale variability respectively. The geochronological reconstruction of the PIS at 35 ka is shown in brown as reference [2]. Red star indicates the position of the marine sediment core MR16-09 PC03 [11].

Supplementary Information References

1. Laskar, J., Robutel, P., Joutel, F., Gastineau, M., Correia, A.C. and Levrard, B. A long-term numerical solution for the insolation quantities of the Earth. Astronomy and Astrophysics, 428(1), pp.261-285 (2004)
2. Davies, B. J., Darvill, C. M., Lovell, H., Bendle, J. M., Dowdeswell, J. A., Fabel, D., ... & Thorndycraft, V. R. The evolution of the Patagonian Ice Sheet from 35 ka to the present day (PATICE). *Earth-Science Reviews*, *204*, 103152 (2020)
3. Kaiser, J., Lamy, F., Arz, H. W., & Hebbeln, D. Dynamics of the millennial-scale sea surface temperature and Patagonian Ice Sheet fluctuations in southern Chile during the last 70 kyr (ODP Site 1233). *Quaternary International*, *161*(1), 77-89. (2007).
4. Heusser, L., Heusser, C., Mix, A., & McManus, J. Chilean and Southeast Pacific paleoclimate variations during the last glacial cycle: directly correlated pollen and δ18O records from ODP Site 1234. *Quaternary Science Reviews*, *25*(23-24), 3404-3415. (2006).

5. De Bar, M. W., Stolwijk, D. J., McManus, J. F., Sinninghe Damsté, J. S., & Schouten, S. A Late Quaternary climate record based on long-chain diol proxies from the Chilean margin. *Climate of the Past*, *14*(11), 1783-1803. (2018).
6. Caniupán, M., Lamy, F., Lange, C.B., Kaiser, J., Arz, H., Kilian, R., Baeza Urrea, O., Aracena, C., Hebbeln, D., Kissel, C. and Laj, C. Millennial‐scale sea surface temperature and Patagonian Ice Sheet changes off southernmost Chile (53 S) over the past~ 60 kyr. *Paleoceanography*, *26*(3). (2011)
7. Lamy, F., Arz, H.W., Kilian, R., Lange, C.B., Lembke-Jene, L., Wengler, M., Kaiser, J., Baeza-Urrea, O., Hall, I.R., Harada, N. and Tiedemann, R. Glacial reduction and millennial-scale variations in Drake Passage throughflow. *Proceedings of the National Academy of Sciences*, *112*(44), 13496-13501. (2015)
8. Ho, S.L., Mollenhauer, G., Lamy, F., Martínez‐Garcia, A., Mohtadi, M., Gersonde, R., Hebbeln, D., Nunez‐Ricardo, S., Rosell‐Melé, A. and Tiedemann, R. Sea surface temperature variability in the Pacific sector of the Southern Ocean over the past 700 kyr. *Paleoceanography*, *27*(4). (2012)
9. Tapia, R., Ho, S.L., Núñez‐Ricardo, S., Marchant, M., Lamy, F. and Hebbeln, D. Increased marine productivity in the Southern Humboldt current system during MIS 2–4 and 10–11. *Paleoceanography and Paleoclimatology*, *36*(4), p.e2020PA004066 (2021)
10. EPICA Community Members. Stable oxygen isotopes of ice core EDML. https://doi.org/10.1594/PANGAEA.754444 (2010).
11. Hagemann, J. R., Lamy, F., Arz, H. W., Lembke-Jene, L., Auderset, A., Harada, N., ... & Tiedemann, R. A marine record of Patagonian ice sheet changes over the past 140,000 years. *Proceedings of the National Academy of Sciences*, *121*(12), e2302983121. (2024).
